# Collaborative development of a digital intervention to support opioid tapering after surgery in primary care: an experience-based co-design study with patients and clinicians

**DOI:** 10.1136/bmjopen-2025-110623

**Published:** 2026-06-30

**Authors:** Neetu Bansal, Christopher J Armitage, Rhiannon E Hawkes, Darren M Ashcroft, Li-Chia Chen

**Affiliations:** 1 Faculty of Biology, Medicine and Health, Centre for Pharmacoepidemiology and Drug Safety, Drug Usage and Pharmacy Practice Group, Division of Pharmacy and Optometry, School of Health Sciences, Manchester Academic Health Science Centre, The University of Manchester, Manchester, UK; 2 Manchester Centre for Health Psychology, School of Health Sciences, The University of Manchester, Manchester, UK

**Keywords:** Primary Care, Pain management, SURGERY

## Abstract

**Abstract:**

**Objectives:**

To co-design a digital intervention (eTAPER) to support safe and timely opioid tapering after surgery in primary care, integrating behavioural theory and stakeholder perspectives.

**Design:**

Qualitative co-design study using two online co-design workshops and one in-person stakeholder engagement event guided by the Experience-Based Co-Design (EBCD) methodology and mapped to the Theoretical Domains Framework. Qualitative data were collected during the two online co-design workshops, while the in-person stakeholder engagement event was used to sense-check and validate the findings from the workshops. No new data were collected during the stakeholder engagement event.

**Setting:**

Two online co-design workshops and one in-person stakeholder event were conducted as part of the EBCD process.

**Participants:**

8 adult post-surgical patients with experience of opioid use and 12 healthcare professionals (general practitioners, clinical pharmacists and anaesthetists) involved in pain management participated. Among patients, 5/8 were female and 5/8 were white British; ages ranged from 25 to 84 years. Among healthcare professionals, 7/12 were female; participants included pharmacists and doctors working across primary care, secondary care and academic settings, with most reporting >10 years of professional experience.

**Results:**

Five key behavioural domains were identified as influencing opioid tapering (environmental context and resources, skills, social/professional role and identity, knowledge and beliefs about consequences). Corresponding behaviour change techniques were embedded within the prototype eTAPER tool. Barriers included poor continuity of care, limited tapering guidance and workforce pressures, while enablers included pharmacist-led reviews and digital education tools. The intervention was refined through iterative workshops to ensure feasibility, usability and alignment with clinical workflows.

**Conclusions:**

This study demonstrates how EBCD can translate lived experience into a scalable digital health intervention. The eTAPER tool offers a theory-informed, co-designed approach to supporting safe opioid tapering after surgery. Further evaluation is needed to assess its effectiveness, usability and transferability across healthcare settings.

**STRENGTHS AND LIMITATIONS OF THIS STUDY**

- The use of Experience-Based Co-Design ensured the active involvement of patients and clinicians in shaping the intervention.
- Integration of behavioural theory (Theoretical Domains Framework and behaviour change techniques) enhanced the intervention’s theoretical grounding and transferability.
- The sample was relatively small and heterogeneous, which may limit transferability beyond similar care contexts.
- Findings are grounded in the UK National Health Service and may require adaptation for use in health systems without comparable digital infrastructure.

## Introduction

 The rising prevalence of opioid misuse following surgery presents a significant public health challenge with profound implications for healthcare systems across developed countries, including the USA, the UK and other developed countries.^1 2^ Advances in surgical care and early hospital discharge have shifted the responsibilities for managing postoperative pain and opioid tapering from secondary to primary care.^3^ However, this transition has exposed a lack of structured support for safe opioid tapering after discharge.

Key challenges include poor communication between care settings, limited guidance for primary care and limited access to specialist support for complex cases.^4 5^ General practitioners (GPs) often feel unequipped to manage tapering in the context of ongoing surgical recovery or unresolved pain, especially when no individualised weaning plan is provided from secondary care. This fragmentation of care not only delays the initiation of tapering but also creates a risk of long-term opioid use.

Although opioid analgesics are generally indicated for only a short duration post-surgery, many patients continue to use them for extended periods.^16^,^8^ Several studies have highlighted concerns regarding excessive opioid prescribing at hospital discharge following surgery. A large multicentre prospective cohort study reported that approximately 30% of patients were prescribed opioids at discharge, and that patients were prescribed more than twice the quantity of opioids they actually consumed within the first 7 days post-discharge.^2^ Similarly, *Daliya et al*, in a retrospective study across 14 National Health Service (NHS) Trusts, found that 21% of patients were prescribed opioids following major surgery.^9^ These findings demonstrate that a substantial proportion of patients continue to receive opioids after discharge, often in quantities exceeding clinical need, highlighting an important opportunity for intervention.

Efforts to improve opioid stewardship in the postoperative period must therefore prioritise safe tapering strategies and continuity of care.^10^ Achieving effective pain control while minimising opioid misuse is critical for improving patient outcomes and mitigating broader societal risks.^11^ Interventions that support safe opioid tapering are increasingly needed to guide clinical practice in the postoperative setting.^12^,^15^ In line with the NHS Long Term Plan’s ‘digital first’ ambition, several digital tools have been introduced to optimise prescribing safety.^16^ One such initiative is the Safety Medication Dashboard (SMASH), a web-based audit and feedback platform that leverages real-time data from the Greater Manchester Care Record.^17^ SMASH has been instrumental in identifying patients at risk of hazardous prescribing based on the nationally recognised PINCER (pharmacist led information technology intervention for reducing *clinically important errors*) indicators.^18 19^ Despite its utility, the platform initially lacked a specific focus on postoperative opioid use, leaving a critical gap in its capacity to address this high-risk area.

To address this challenge, the eTAPER intervention was developed for integration within the Safety Medication Dashboard (SMASH), a pharmacist-led electronic audit and feedback platform used in primary care to support medication safety reviews.^18^ The intervention is designed to identify patients prescribed opioids following surgery and prompt timely review and opioid tapering in primary care, rather than at the point of initial prescribing. The eTAPER intervention is a clinician-facing digital decision support tool and is not directly accessible to patients. Patients were involved in the co-design process to ensure the tool reflects lived experience of postoperative opioid use and to optimise how tapering advice is communicated in clinical consultations. Their input helped enhance acceptability, clarity and support for shared decision-making. eTAPER was developed using Experience-Based Co-Design (EBCD), a methodology that incorporates service users’ experience to inform healthcare improvement.^20^ While EBCD has demonstrated value in the development of digital health tools and chronic disease management interventions,^21^,^23^ it remains underused in the context of opioid stewardship.

This study builds on earlier phases of the EBCD process, which identified key barriers to opioid tapering after surgery.^24^ These findings informed the initial development of the eTAPER intervention. In the present study, we undertook the final co-design phase to refine the intervention. The same patient participants were involved across both phases, allowing continuity and iterative refinement based on their experiences.

**Figure 1:**
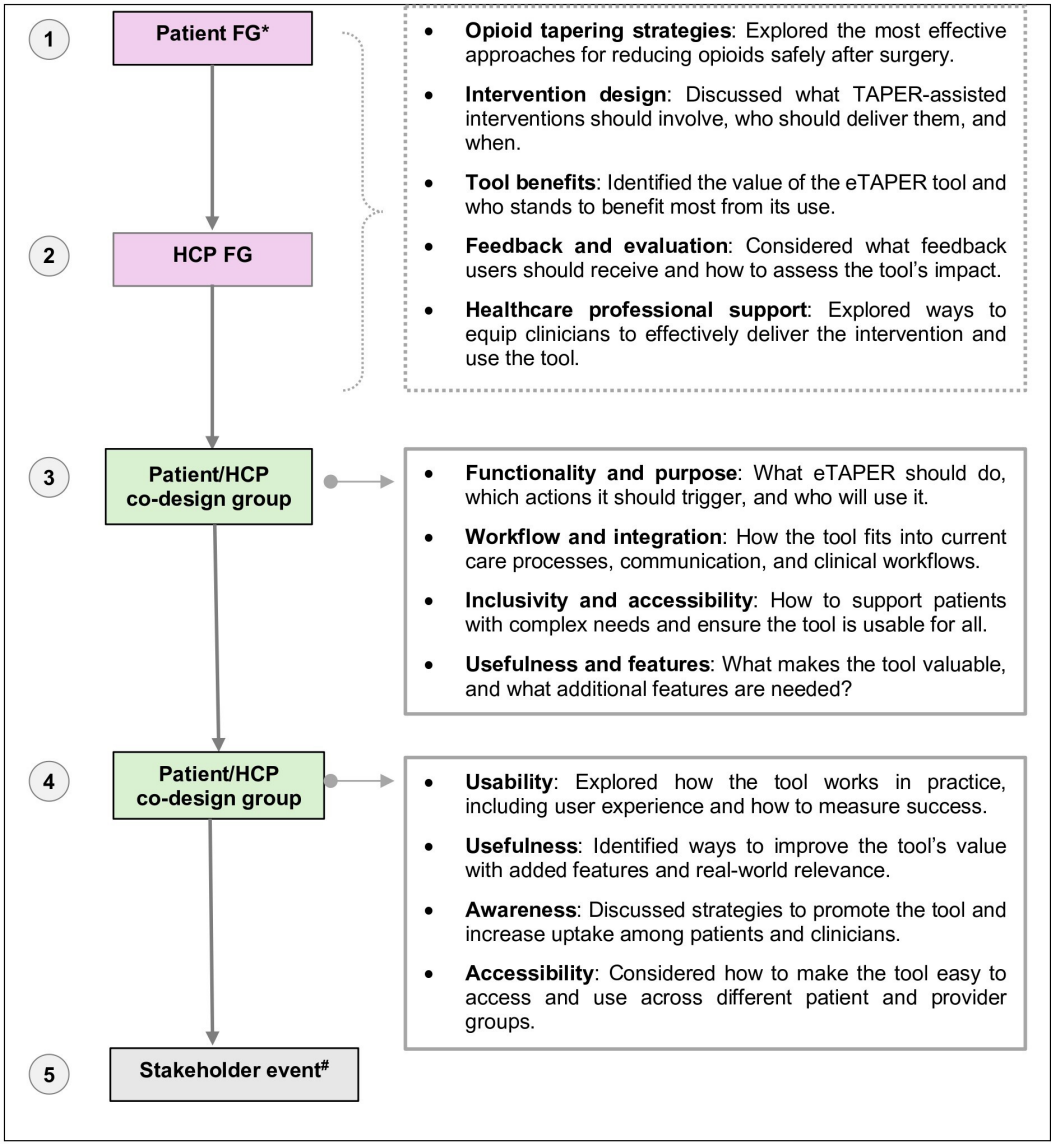
The EBCD process of developing the TAPER tool and themes explored in the focus groups. *In-person event; ^#^hybrid event. This paper focuses on the results from sessions 3–5. EBCD, Experience-Based Co-Design; FG, focus group; HCP, healthcare practitioners.

This study presents findings from the final phase of the EBCD process focused on the development of the eTAPER intervention (figure 1). It draws on insights from two joint co-design workshops involving patients and healthcare professionals, aimed at refining the intervention and ensuring its feasibility, acceptability and alignment with primary care workflows. These discussions explored the barriers and facilitators to effective tapering, identified relevant behaviour change techniques (BCTs) and informed the development of the eTAPER intervention designed to improve the management of opioid tapering in postoperative care.

## Methods

### Study design

This study employed a qualitative design guided by the principles of the EBCD approach to inform the development of the eTAPER intervention (online supplemental file 1). Reporting followed the Consolidated criteria for Reporting Qualitative research (COREQ) checklist for qualitative research. Completed checklists are provided in online supplemental file 2. EBCD was selected to ensure the intervention was grounded in the lived experiences of both patients and healthcare professionals, consistent with patient-centred service improvement.^20 22 23^

The study followed a multistage EBCD process (figure 1). Initially, two separate focus groups were conducted with patients and healthcare professionals to explore experiences of opioid prescribing and tapering following surgery (stages 1–2).^24^ These discussions were analysed using the Theoretical Domains Framework (TDF) to identify behavioural determinants influencing current practices.^25^ Findings from the patient session informed the development of a ‘trigger film’ which captured key patient narratives related to opioid use. Patients subsequently viewed the trigger film and confirmed it reflected their experiences, thereby validating the qualitative findings. This film was later used as a stimulus to promote empathy and shared understanding during joint co-design sessions.^26^

Subsequently, two online co-design workshops were held, bringing together patients and healthcare professionals to collaboratively identify intervention priorities and inform the iterative development of the eTAPER prototype. The first session focused on clarifying the tool’s intended functions, identifying key actors and workflows and exploring support needs for implementation in clinical practice. Insights from this session informed the initial design of the prototype. The second session refined the prototype further, with discussions centred on usability, relevance, accessibility and strategies for promoting awareness among users. Participants stressed the importance of integrating the tool into routine workflows without increasing administrative burden (figure 1).

A subsequent in-person stakeholder engagement event (stage 5) was conducted to present and sense-check emerging findings; however, no data from this event were included in the formal analysis. Attendees included clinicians, patients, researchers and policymakers.

All participants in the online co-design workshops provided informed consent and were assured of confidentiality and anonymity throughout the research process.

### Participants

Patient participants were adults (aged 18 and over) who had undergone surgery and received opioid prescriptions for postoperative pain, as well as healthcare professionals involved in post-surgical pain management within the NHS, including general practitioners, pharmacists and anaesthetists. Participants were recruited from 9 February 2024 until 8 March 2024. Efforts were made to ensure diversity in professional backgrounds and care settings by purposively sampling participants from a range of roles. This approach aimed to capture a broad spectrum of perspectives on opioid tapering across the care continuum.

Healthcare professionals were selected to reflect those most involved in ongoing opioid prescribing and tapering following hospital discharge, particularly within primary care settings where responsibility for pain management is often transferred. Pharmacists, including those working in general practice, were purposively included due to their central role in medicines optimisation and their interaction with prescribing safety platforms such as SMASH, within which the eTAPER intervention is embedded. While surgeons play an important role in initiating postoperative opioid prescribing, their involvement in longer-term opioid management is typically limited; therefore, the study prioritised professionals involved in continuity of care after discharge. Nevertheless, anaesthetists were included to provide perspectives from secondary care pain management.

Patients were recruited via social media platforms (Twitter/X, LinkedIn, Facebook) and through collaboration with the pain charity Pain Concern, which promoted the study through its networks. A purposive sample of healthcare professionals was recruited using professional contacts, online platforms and snowball sampling techniques. All participants provided written informed consent prior to participation. Recruitment materials emphasised the voluntary nature of the study and provided detailed information about its aims and procedure. Consent was obtained for audio recording, and all sessions were transcribed verbatim by a professional transcription service bound by confidentiality agreements.

### Data collection

Data collection took place between March 2024 and July 2024. The two online co-design workshops were conducted using video conferencing software. Two trained moderators facilitated each session (NB and L-cC). Given the relatively large number of participants per session, participants were divided into smaller breakout rooms (typically four to six participants per group). Breakout rooms were purposively composed to include a mix of patients and healthcare professionals, and each room was supported by a facilitator to guide discussion and ensure consistency across groups.

The purpose of the breakout rooms was to enhance interaction and depth of discussion rather than to conduct separate focus groups. Following the breakout discussions, participants reconvened in a plenary session where key points from each group were shared and discussed collectively, allowing for comparison and refinement of ideas across groups. Therefore, each workshop was treated analytically as a single co-design session rather than multiple independent focus groups.

At the beginning of each session, participants were briefed on the study’s aims and ground rules, with an emphasis on confidentiality and respectful dialogue. Participants were informed about the confidentiality and anonymity of their contributions. Discussion topic guides were developed to steer the co-design sessions (online supplemental file 3 and 4). The guides were informed by earlier focus group findings from stages 1–2 (figure 1) and aimed to elicit participants’ views on practical and theoretical aspects of the intervention.

Each online co-design workshop lasted approximately 120 min. The sessions were audio-recorded and transcribed verbatim by a professional transcription service bound by confidentiality agreements. All transcripts were pseudonymised before analysis to protect participants’ identities.

### Analysis

Thematic analysis was used to analyse the data, with the initial coding conducted inductively to develop a coding framework and identify themes.^27^ The lead researcher (NB) performed the initial inductive coding and reviewed it in collaboration with a second researcher (REH) to develop a coding framework. The themes identified were then deductively mapped to the TDF (online supplemental file 5) to identify key behavioural influences.

The TDF was selected as it provides a comprehensive, theory-informed framework that synthesises constructs from multiple behaviour change theories into a set of domains relevant to understanding healthcare behaviours.^25^ Its use is well established in implementation research and is particularly suited to identifying determinants of complex behaviours, such as opioid prescribing and tapering across care transitions. Applying the TDF enabled the research team to move beyond descriptive themes and systematically interpret the underlying behavioural mechanisms influencing both patient and clinician actions.

The mapping of themes to TDF domains was conducted independently by two researchers (NB and REH), with discrepancies resolved through discussion and input from a third author (CJA). Microsoft Excel was used to support data management and the application of the framework approach, which enabled both emergent and theory-driven themes to be analysed in parallel.^28^ This approach was chosen as it enabled both predetermined and emergent issues to be explored in depth while using the TDF as an explanatory framework.^28^

The TDF consists of 14 domains encompassing a range of individual, social and environmental influences on behaviour.^29^ Domains identified in this study were used to inform the selection of appropriate BCTs using the Theory and Techniques Tool.^30^ This tool facilitates the systematic linking of behavioural determinants with evidence-based intervention strategies.

### Researcher positioning

The lead researcher (NB) is a female clinical academic pharmacist with a PhD and training in qualitative methods and experience in perioperative medicines optimisation. Her professional background may have influenced both participant engagement and interpretation of the data. To mitigate potential confirmation bias, a second coder (REH), trained in health psychology, independently reviewed coding and contributed to framework development; discrepancies were resolved through discussion within a multidisciplinary team (pharmacy, health psychology, pharmacoepidemiology). Participants were briefed on the study aims, NB’s role and the purpose of the sessions; no prior personal relationships existed with patient participants, and any pre-existing professional acquaintance with a small number of healthcare practitioner participants arose only via recruitment networks and did not include supervisory relationships. Facilitation used open-ended prompts and a standardised guide to minimise leading questions and encourage diverse perspectives.

### Patient and public involvement

Patients were actively involved throughout this study using the EBCD approach. Post-surgical patients participated in focus groups (stages 1–2) to identify priorities, contributed to the development of a trigger film to support shared understanding and worked alongside clinicians in co-design workshops to shape the intervention (stages 3–4). Patients also took part in a stakeholder engagement event (stage 5) to reflect on the acceptability and feasibility of the prototype. The research team will continue to involve patients in future stages of evaluation and implementation.

## Results

### Characteristics of participants

A total of eight patient participants (aged 25–84 years, median 49.5 years) with a history of postoperative opioid use took part. Participants were 5/8 female (62.5%) and 3/8 male (37.5%); 5/8 (62.5%) identified as white British, with the remaining participants identifying as British Asian or British African, representing a range of ethnic backgrounds and surgical experiences. The study included 12 healthcare professionals comprising pharmacists and doctors working across primary care, secondary care, commissioning and academic settings. Participants were 7/12 female (58.3%) and 5/12 male (41.7%); 8/12 (66.7%) were white British and 4/12 (33.3%) were British Asian, with most having >10 years of professional experience.

To ensure participant anonymity while enabling clear attribution of perspectives, each quoted contribution in the findings is coded using an alphanumeric identifier. The letter denotes the participant’s role, ‘P’ for patients and ‘H’ for healthcare professionals, followed by a number corresponding to their unique contributions (eg, P1, H1). This system facilitates the traceability of individual voices while maintaining confidentiality.

Analysis of the discussions of two online co-design workshops identified five primary theoretical domains that were most frequently referenced: environmental context and resources (164 of 376 coded instances), skills (61), social/professional role and identity (48), knowledge (38) and beliefs about consequences (17) (table 1). These domains informed the thematic structure of the analysis and the derivation of relevant BCTs, which are presented in the subsequent sections.

**Table 1:** Theoretical domains identified from the two co-design sessions

Theoretical domain	Relative frequency
Environmental context and resources	High
Skills/knowledge	High
Social/professional role and identity	Moderate–high
Belief about consequences	Moderate
Social influences/intentions	Low–moderate
Other behavioural domains	Low

### Environmental context and resources

Key themes within this domain included communication, workflow integration and the development of effective pain management strategies. Participants emphasised the need for standardised practices to ensure consistency in opioid tapering and patient education. Suggestions included the use of multilingual translated leaflets, plain language and visual aids, with a focus on tailoring information to specific procedures and varying levels of health literacy. Recommendations to enhance communication included text message reminders for upcoming reviews and educational messaging. The flexibility of consultation formats, face-to-face, telephone and video calls was also noted, particularly when integrated with patient records.

It’s not just about doing a set number of reviews—we need to ensure quality and accessibility in the information we provide. (P1)

It’s quite easy to switch to a video call in general practice now because it’s all linked to the patient notes. (H7)

Effective implementation of the eTAPER tool was seen as requiring seamless integration into clinical workflows without increasing the administrative burden. Healthcare professionals noted that workforce capacity is already stretched, and tools perceived as duplicative or burdensome are unlikely to be adopted. Community pharmacists were identified as a valuable resource in supporting opioid tapering, particularly in providing follow-up, potentially easing pressure on general practice.

For the tool to be effective, it must be embedded within routine clinical practices and avoid being perceived as a procedural or tokenistic task. Rather than functioning as a checklist, it should actively support clinical decision-making and promote sustained patient engagement. Given that opioid tapering is a progressive process, longitudinal follow-up was considered essential. The tool should enable ongoing monitoring rather than a single-point review, incorporating mechanisms, such as patient filtering systems to facilitate targeted and timely follow-up.

Multidisciplinary team involvement, spanning nurses, physiotherapists and other allied health professionals was seen as essential for coordinated care. Ensuring all team members are aware of a patient’s opioid tapering plan was viewed as key to embedding the intervention into routine practice and establishing clear accountability for its use.

We need a structured plan or guidance from the tool. (P2)

It’s essential that the workflow remains manageable. (H3)

Participants discussed the need for a balanced approach that addresses acute post-surgical pain while reducing the risk of prolonged or unnecessary opioid use. The risks associated with so-called ‘weaker’ opioids such as tramadol and codeine were also raised, noting that these medications present unique challenges around dependence and suboptimal analgesia. They suggested that the eTAPER tool must be designed to guide healthcare professionals in recognising these challenges and ensuring that opioid use is optimised based on individual patient needs.

A lot of patients may be receiving weaker opioids like tramadol and codeine, which create their own challenge and problems. (H5)

Rather than applying a generalised approach to pain management, participants emphasised the importance of a targeted focus on postsurgical opioid prescribing. The tool should support effective short-term pain relief while facilitating timely and appropriate tapering.

The tool needs to be focused on optimising post-surgical opioids. (P4)

### Knowledge

A central theme within this domain was the need to enhance awareness and understanding of tools and guidelines relevant to opioid tapering. Healthcare professionals highlighted the importance of becoming familiar with SMASH, the existing electronic platform supporting medication safety interventions, as this is a key step toward embedding eTAPER into routine clinical workflows. Ensuring access to practical resources, such as opioid tapering guidelines, evidence-based decision aids and patient information leaflets, was seen as essential for increasing confidence and competence in managing opioid-related care. This domain emphasises the foundational knowledge required to support safe, informed and standardised practice.

I think it’s called the McDonald’s theory—if you put someone in front of the machines, they can make a burger fairly easily without extensive training. (P7)

One thing that might be useful is a bit of user-generated content—like aide-memoires and cheat sheets. (H9)

### Belief about consequences

The key theme highlighted within this domain was the importance of establishing clear criteria for evaluating the success of the eTAPER tool. Participants emphasised that a key outcome should be the timely discontinuation of opioids, particularly among opioid-naive patients. Continued opioid use beyond 30 days post-surgery was viewed as a red flag and a potential indicator of suboptimal tapering support. Therefore, a reduction in opioid prescribing within a defined postoperative period was identified as a critical measure of the tool’s effectiveness. Participants agreed that eTAPER’s impact should ultimately be assessed by its ability to support patients in discontinuing opioids safely and efficiently following acute surgical pain.

Patients opioid naive should not still be on opioids at 30 days post-op. That’s quite a significant event, I think if that happens. (H10)

### Social/professional role and identity

A multidisciplinary approach to postoperative pain management was consistently emphasised, with participants advocating for collaborative care involving pharmacists, surgeons and other healthcare professionals. Pharmacist-led medication reviews and greater surgeon involvement in post-discharge opioid management were seen as key strategies to improve tapering outcomes. Optimising existing pathways, such as enhancing communication with community pharmacists and improving discharge counselling was also identified as critical.

Given general practice time constraints, participants recommended redistributing prescribing responsibilities across the primary care team, including advanced clinical practitioners, physiotherapists, nurses and pharmacists. A holistic model of care involving physiotherapists, occupational therapists and psychologists to address both physical and psychological aspects of pain was strongly endorsed.

Patients require time; investing in psychologists and more GPs is essential. (H8)

Clearly defined roles and responsibilities in opioid tapering were considered essential to ensure continuity and coordination across care settings. A consistent point of contact throughout the tapering process was viewed as crucial to maintaining patient engagement and preventing gaps in care during transitions from secondary to primary care.

eTAPER should be mentioned in secondary care to patients at the point of discharge. (H11)

Trust between healthcare professionals and patients emerged as a central theme. Participants highlighted the importance of compassionate communication and reassurance to foster adherence to tapering plans. Introducing eTAPER during hospital discharge was seen as an essential step to build patient understanding and trust early in the process.

It’s a very good point in regard to who has the responsibility, whether it’s the patient or a named individual. From a patient perspective, it’s that trust element, ie, for myself, I only see two GPs in my practice. I will not see any others. That’s because those two, I trust in regard to not to mess about with…because I’m on polypharmacy. (P3)

Participants also suggested that trust could be undermined if digital tools were perceived as monitoring or restricting prescribing without adequate clinical context. Ensuring that eTAPER supports, rather than replaces, clinical judgement was therefore considered essential. This was particularly important in the context of increasing regulatory scrutiny around opioid prescribing, where clinicians must balance patient-centred care with accountability and safe prescribing practices.

With something like this, if you build that trust and that relationship, then it’s going to be much easier to look for other ways of managing pain, rather than just giving out another prescription when it’s not necessarily the best thing. Also, the SMASH board, there needs to be one for professionals and an easier read for patients to show the benefits of the SMASH tool and why it’s beneficial to come off the opiates. (P5)

Like we discussed earlier, we don’t want the eTAPER tool, we discussed this last week, to look like a checklist and that’s where it wouldn’t work. It needs to be an overall, holistic review considering everything. (H6)

One participant highlighted the importance of continuity in fostering trust:

I actually think it’s generally within our power to give continuity but it does take a little bit more time and effort. It’s much easier to say, ‘oh, book another appointment’, and then you end up with a different doctor or a different pharmacist. I think it is possible and I think it’s super important. With something like this, if you build that trust and that relationship, then it’s going to be much easier to look for other ways of managing pain, rather than just giving out another prescription when it’s not necessarily the best thing. (H9)

### Skills

This domain is centred on equipping healthcare professionals and patients with the skills required for effective opioid tapering and promoting alternative pain management strategies. Participants emphasised the importance of structured education for both groups to ensure that patients are well-informed about tapering processes and that healthcare providers are adequately prepared to support them. Providing patients with accessible, standardised information was seen as essential for improving adherence and reducing unnecessary opioid use. A consistent recommendation was the development of an information leaflet outlining recovery timelines, tapering goals and key milestones to align expectations across patients, carers and clinicians.

I actually think it could be really, really useful, this tool, because it gives a chance for dialogue between healthcare professional and patient about this important issue. (P8)

Everyone receiving the same message is crucial. Perhaps an initial text could be used to prime patients, followed by a leaflet highlighting what needs to be achieved post-surgery, along with the aims and expected timelines so that everyone is aligned. This would be a simple way to keep things streamlined and consistent. (P5)

Healthcare professionals expressed the need for targeted training and embedding decision-support tools to guide appropriate opioid tapering decisions. Integrating evidence-based guidance within eTAPER was viewed as a way to enhance its effectiveness. Clear communication around pain trajectories, side effect management and practical planning (eg, return to work or caregiving duties) was also emphasised.

What I’m asking is I’d appreciate there will be more clear instructions, side-effect management, and direct communication with the GPs. (P9)

Participants also emphasised the value of promoting non-pharmacological strategies, such as physiotherapy and cognitive behavioural techniques, to support patients in managing pain beyond opioids. Embedding these resources into eTAPER was seen as essential for delivering a comprehensive, patient-centred approach to tapering.

### Behaviour change techniques identified from theoretical domains

Overall, five core BCTs were identified as critical to facilitating effective opioid tapering through the eTAPER tool. These included prompts and cues, provision of practical social support, shaping knowledge, highlighting the natural consequences of opioid use and encouraging behavioural practice and rehearsal (table 2). Collectively, these techniques provide a structured foundation to support behaviour change among both patients and healthcare professionals, enhancing the tool’s potential to enable safe and sustained opioid reduction in clinical practice.

**Table 2:** Behaviour change techniques identified from the co-design sessions

Behaviour change techniques	Example of intervention
Prompts/cues	Providing timely reminders and notifications to encourage adherence to opioid tapering plans and information about health consequences.
Social support (practical)	Facilitating support from healthcare professionals and caregivers to guide patients through the tapering process.
Shaping knowledge (category)	Enhancing patient and clinician understanding of opioid reduction strategies through structured education and decision-support tools.
Natural consequences (category)	Emphasising the potential benefits of opioid reduction, such as improved overall health and reduced dependence, to motivate behaviour change.
Behavioural practice/rehearsal	Encouraging patients to engage in alternative pain management strategies, such as physiotherapy or cognitive behavioural techniques, to reinforce non-pharmacological coping mechanisms.

## Discussion

This study developed a co-designed digital intervention (eTAPER) to support safe and timely opioid tapering following surgery. Grounded in behavioural theory and integrated into existing clinical workflows, the intervention responds directly to key challenges identified by patients and healthcare professionals, including lack of tapering guidance, poor continuity of care and limited patient education.^24 31^ These findings reinforce the value of theory-informed and implementation-ready interventions and align with previous studies emphasising patient-centred care and interprofessional collaboration in optimising postoperative pain management and opioid stewardship.^4 32 33^

Digital interventions to support opioid optimisation and tapering are increasingly being developed, including patient-facing applications, electronic prescribing alerts and clinical decision support tools.^34 35^ Many of these interventions focus on discrete components of care, such as patient education, pain self-management or prescribing alerts, and are often implemented either at the point of prescribing or as standalone tools. In contrast, the eTAPER intervention has been designed as a clinician-facing, primary care-integrated tool embedded within an existing prescribing safety platform (SMASH). This enables identification of patients prescribed opioids following surgery and supports timely review and tapering within routine clinical workflows. Notably, eTAPER specifically targets the transition between secondary and primary care, a recognised high-risk period for ongoing opioid use that is often not addressed by existing digital tools.

Patient involvement in the co-design process also has important implications for trust, which is a critical determinant of engagement with digital health interventions. By incorporating patient perspectives throughout development, eTAPER was shaped to reflect patient priorities, concerns and lived experiences, which may enhance transparency, perceived relevance and trust in the tool. This is particularly important in the context of opioid tapering, where patients may be sensitive to perceived reductions in medication or concerns about being monitored or restricted.

However, trust may be undermined if digital tools are perceived as replacing clinical judgement or acting as surveillance mechanisms. Future iterations of eTAPER should therefore prioritise clear communication about the purpose of the tool, emphasising its role in supporting, rather than directing, clinical decision-making. Enhancing continuity of care, enabling personalised tapering plans and ensuring that clinicians retain flexibility to adapt recommendations will be essential. Ongoing co-refinement with patients and healthcare professionals, alongside transparent messaging and training, will be key to strengthening trust and supporting sustained engagement with the intervention.

Five core behavioural domains, environmental context and resources, knowledge, skills, social/professional role and identity and beliefs about consequences were identified through stakeholder data and mapped to corresponding BCTs. These domains not only informed the content and structure of the eTAPER intervention but also guided the development of implementation strategies to ensure successful uptake in practice. For example, prompts and cues were used to address environmental context, training and information materials supported knowledge and skills and role clarification helped reinforce professional identity and accountability in opioid tapering. By integrating these theory-driven components into the design process, the eTAPER tool was iteratively refined to reflect real-world challenges, enhance usability and support both clinicians and patients through the tapering process sustainably and practically.

The findings also provide clear direction for implementation. Successful uptake will depend on a combination of system-level processes and local engagement. Stakeholders emphasised the need for structured training and education to ensure that GPs, pharmacists and wider healthcare teams are confident in managing tapering conversations, addressing side effects and navigating the features of the intervention. Implementation should be supported by proactive use of the SMASH dashboard (eg, weekly checks by GPs or GP pharmacists) and reactive alerts triggered by opioid prescribing data, which can prompt text messages or app-based notifications to patients, as well as communication with healthcare providers. To promote adoption, participants suggested enhancing usability, accessibility and awareness, as well as developing a clear communications plan to introduce eTAPER to both patients and professionals as part of a system-wide strategy for safer opioid tapering.

A key strength of the eTAPER intervention is its development through an EBCD approach. This process enabled the incorporation of lived experiences from both patients and healthcare professionals, informing features that extend beyond those typically captured in existing digital tools. In addition, the integration of BCTs, identified through prior systematic review and mapped to the TDF, represents a theoretically informed approach to intervention design. This contrasts with many existing digital tools, which may lack an explicit behavioural framework underpinning their development. Taken together, these features suggest that eTAPER offers a novel contribution by combining digital infrastructure, behavioural theory and co-designed insights to address a critical gap in postoperative opioid management.

However, several limitations should be considered. The relatively small and heterogeneous sample, comprising 8 post-surgical patients and 12 clinicians, may limit the representativeness of the findings, although the insights generated are likely to be transferable to similar care contexts. As with most qualitative co-design studies, self-selection bias and reliance on self-reported data may have influenced the findings. The use of an online format facilitated participation but may have constrained natural interaction and limited the capture of non-verbal cues, particularly in mixed stakeholder workshops where power dynamics required careful facilitation. Furthermore, while the intervention is designed for integration within the SMASH platform in Greater Manchester, its reliance on established digital infrastructure may limit transferability to settings without comparable systems.

Finally, while the formal study has concluded, there is an opportunity for continued engagement with participants. Several expressed interest in further collaboration, and involving them in implementation, evaluation or dissemination efforts would strengthen the person-centred ethos of the project and support sustainable integration into practice.

This study illustrates how co-designed digital interventions can bridge critical discontinuities in post-discharge care. The eTAPER model also supports broader policy goals around safe opioid prescribing, medicines optimisation and digital health innovation in primary care.^36^,^38^ It may inform future commissioning decisions and national strategies aimed at reducing long-term opioid-related harm.

To improve usability and implementation, future iterations of the eTAPER intervention should include training components for clinicians on tapering strategies, managing patient queries and recognising adverse effects. Future iterations should also explore integration at the presurgical stage to enable earlier identification and planning and include functionality that supports seamless information sharing across care settings to promote continuity. Usability testing and co-refinement with end users will be critical in ensuring the tool is accessible, acceptable and adaptable across diverse patient populations and clinical environments.

## Conclusion

This study presents the development of eTAPER, an innovative, co-designed intervention aimed at optimising postoperative opioid use through a structured, patient-centred and provider-centred approach. By employing the EBCD, we were able to directly integrate the insights and needs of both patients and healthcare professionals into a prototype that addresses the complexities of opioid tapering in the post-surgical setting.

While the study confirms the feasibility and acceptability of the co-design approach in creating digital health interventions, it also identifies challenges that must be addressed in future iterations. These include ensuring robust inter-professional communication, providing adequate training to healthcare providers and adapting the tool for diverse clinical settings.

In summary, the eTAPER tool represents a promising step forward in postoperative pain management and opioid optimisation. Its collaborative design and integration of behaviour change strategies position it as a valuable resource in addressing the ongoing challenges of opioid prescribing and patient care.

## Supplementary material

10.1136/bmjopen-2025-110623 online supplemental file 1

## Data Availability

All data relevant to the study are included in the article or uploaded as supplementary information.
