# Supplementary material for: Collaborative development of a digital intervention to support opioid tapering after surgery in primary care: an experience-based co-design study with patients and clinicians
Source: BMJ Open. 2026 Jun 30;16(6):e110623. doi: 10.1136/bmjopen-2025-110623 (PMC13331231; doi:10.1136/bmjopen-2025-110623)
Supplement: online supplemental file 1 [file bmjopen-16-6-s001.docx]

# Online supplementary material

Online supplement 1. Defining the experience-based co-design methodology

| **Stage** | **Description** |
| --- | --- |
| **Identifying the issue** | Initial scoping of the problem through literature review and stakeholder input. |
| **Engagement & recruitment** | Patients and healthcare professionals invited to participate in co-design sessions. |
| **Capturing experiences** | Collection of experiences through interviews, focus groups, and 'trigger films' that highlight key issues. |
| **Mapping emotional journeys** | Patients review collected experiences, mapping emotional responses to pain management and opioid use. |
| **Prioritisation of key issues** | Stakeholders collaboratively identify and rank the most pressing issues requiring intervention. |
| **Co-design workshops** | Patients and healthcare professionals work together to develop solutions and refine interventions. |
| **Prototype development** | Refinement of interventions and integration of behaviour change techniques into a structured tool. |
| **Celebration event** | Summarise the impact of the intervention, including the finalised eTAPER tool and its integration into practice. |
| **Implementation & testing** | Pilot testing of the intervention in clinical settings with iterative feedback loops. |
| **Sustained improvement** | Embedding the co-designed intervention into practice and evaluating long-term impact. |

# Online supplement 2

# COREQ (COnsolidated criteria for REporting Qualitative research) Checklist

A checklist of items that should be included in reports of qualitative research. You must report the page number in your manuscript where you consider each of the items listed in this checklist. If you have not included this information, either revise your manuscript accordingly before submitting or note N/A

| **Topic** | **Item no.** | **Guide question / description** | **Reported on Page No** |
| --- | --- | --- | --- |
| **Domain 1: Research team and reflexivity** | | | |
| *Personal characteristics* | | | |
| *Interviewer/facilitator* | 1 | Which author/s conducted the interview or focus group? | 8 |
| *Credentials* | 2 | What were the researcher’s credentials? E.g. PhD, MD | 10 |
| *Occupation* | 3 | What was their occupation at the time of the study? | 9 |
| *Gender* | 4 | Was the researcher male or female? | 10 |
| *Experience and training* | 5 | What experience or training did the researcher have? | 9 |
| *Relationship with participants* | | | |
| *Relationship established* | 6 | Was a relationship established prior to study commencement? | 9 |
| *Participant knowledge of*  *the interviewer* | 7 | What did the participants know about the  researcher? e.g. personal goals, reasons for doing the research | 9 |
| *Interviewer characteristics* | 8 | What characteristics were reported about the  inter viewer/facilitator? Bias, assumptions, reasons and interests in the research topic | 9 |
| **Domain 2: Study design** | | | |
| *Theoretical framework* | | | |
| *Methodological orientation and Theory* | 9 | What methodological orientation was stated to underpin the study? e.g. grounded theory, discourse analysis, ethnography, phenomenology, content analysis | 8 |
| *Participant selection* | | | |
| *Sampling* | 10 | How were participants selected? e.g. purposive, convenience, consecutive, snowball | 7 |
| *Method of approach* | 11 | How were participants approached? e.g. face-to-face, telephone, mail, email | 7 |
| *Sample size* | 12 | How many participants were in the study? | 7 |
| *Non-participation* | 13 | How many people refused to participate or dropped out? Reasons? | N/A |
| *Setting* | | | |
| *Setting of data collection* | 14 | Where was the data collected? e.g. home, clinic, workplace | 8 |
| *Presence of non- participants* | 15 | Was anyone else present besides the participants and researchers? | 10 |
| *Description of sample* | 16 | What are the important characteristics of the sample? e.g. demographic data, date | 10 |
| *Data collection* | | | |
| *Interview guide* | 17 | Were questions, prompts, guides provided by the authors? Was it pilot tested? | 8 |
| *Repeat interviews* | 18 | Were repeat inter views carried out? If yes, how many? | N/A |
| *Audio/visual recording* | 19 | Did the research use audio or visual recording to collect the data? | 8 |
| *Field notes* | 20 | Were ﬁeld notes made during and/or after the interview or focus group? | 8 |
| *Duration* | 21 | What was the duration of the inter views or focus group? | 8 |
| *Data saturation* | 22 | Was data saturation discussed? | N/A |
| *Transcripts returned* | 23 | Were transcripts returned to participants for comment and/or correction | N/A |
| **Domain 3: analysis and ﬁndings** | | | |
| *Data analysis* | | | |
| Number of data coders | 24 | How many data coders coded the data? | 9 |
| Description of the coding  tree | 25 | Did authors provide a description of the coding tree? | N/A - analysis was guided by the Theoretical Domains Framework |
| Derivation of themes | 26 | Were themes identiﬁed in advance or derived from the data? | 9 |
| Software | 27 | What software, if applicable, was used to manage the data? | 9 |
| Participant checking | 28 | Did participants provide feedback on the ﬁndings? | 6 |
| *Reporting* | | | |
| Quotations presented | 29 | Were participant quotations presented to illustrate the themes/ﬁndings? | 9 |
| Data and ﬁndings consistent | 30 | Was each quotation identiﬁed? e.g. participant number | N/A |
| Clarity of major themes | 31 | Was there consistency between the data presented and the ﬁndings? | 10-15 |
| Clarity of minor themes | 32 | Is there a description of diverse cases or discussion of minor themes? | N/A |

Developed from Tong A, Sainsbury P, Craig J. Consolidated criteria for reporting qualitative research (COREQ): a 32-item checklist for interviews and focus groups. *International Journal for Quality in Health Care*. 2007. Volume 19, Number 6: pp. 349 – 357

Online supplement 3. Focus group schedule for the co-design workshop (1)

Developing An Intervention to Optimise Acute Pain and Opioid Use After Surgery

**Joint Patient and Healthcare Professional Focus Group Schedule – Session 3**

**Opening**

(Introduction, briefing the purposes of the focus group, set up the ground rules)

Good morning, everyone. Welcome to our third experience-based co-design session. I am delighted we will be working together today as a team of patients and healthcare professionals to further develop the features of the eTAPER tool.

***(Summary of the previous meeting)***

The trigger film will last 12 minutes, followed by a 5-minute presentation and up to 10 minutes of discussion on themes that emerge from the trigger video film.

In our last meeting, each group, comprising patients and healthcare professionals, focused on post-operative surgical pain management challenges. We discussed strategies to enhance the patient's surgical journey.

Before delving into today's discussion, let's revisit our previous sessions. We'll watch a short video depicting patients' experiences with post-surgery pain management using opioids. Afterwards, I'll provide a concise summary of our past discussions.

First, let me share this video with you. Play the trigger film video (12 minutes). I hope this video has highlighted some critical issues of patients' experiences and views. (Share screen of PowerPoint slide show.)

In summary, patients highlighted some challenges in surgical pain management and opioid use… (present summaries on slide 1).

Also, healthcare professionals felt … (present summaries on slide 2).

I hope these summaries capture all the critical points. Are there any burning points or views that you would like to share and discuss at this moment? We have a couple of minutes.

***(Aim of this meeting)***

In this and the next sessions, we will work together to develop the tool's features. Following our discussion today, we will further refine the draft prototype, which will be ready for discussion again in the next session.

Before we start the discussion, I would like to set the theme for today's discussions by introducing the current features of the draft prototype of eTaper (present slide 3).

The key discussion focus for today is … (present slide 4).

***(Schedule and housekeeping)***

To facilitate effective conversations, we will divide into two breakout rooms, each led by two facilitators.

Facilitators will help with the focus group discussions, take notes and manage the time.

If you need a break during the discussion, please inform facilitators using the chat box.

The facilitator will remain impartial throughout the event.

***(Confidentiality and anonymity of recording)***

Throughout our discussion, we will be recording audio.

Your honesty is paramount, and there are no right or wrong answers. Please be assured all conversations will be treated with utmost confidentiality and anonymity. Do not disclose identifiable information outside this focus group.

***(Ground rules)***

Before we begin, let's agree on some ground rules:

We kindly ask that we take turns speaking to ensure a clear audio recording and allow others to express their opinions even if you disagree with them.

If you want to share your views, please use the '**raise your hand**' function and respect the discussion's confidentiality.

Please also express your views verbally to enable audio recording.

Thank you for being a part of this vital conversation. Your insights will make a real difference.

***(Split into breakout rooms)***

Now, we will split into two breakout rooms for the main discussion for **60 minutes**, and then we will get back together in the main room at **11:40** to wrap up our discussions today. See you back at **11:40**.

Discussion

Hello, and welcome to the discussion of this breakout room. My name is [XXXX], and I will facilitate the discussions. Together with me is [XXX] to help with this session. We have [XXXXXX] (name the participants) in this group.

**Ice breaking / Introduction**

Introduction. I think it would be useful if we got to know each other. Some of you have met. Could we go around the room introducing ourselves? For patients (name, type of surgery, current pain medication). For healthcare professionals (name and roles)

**Defining the purpose and actions of the eTAPER Tool**

(Discuss and reach agreement on the primary purpose and key actions of the eTAPER tool.)

First, based on the current feature of the draft eTaper, I would like to know your views on "what should be the main purpose of the eTAPER tool" and "what actions should it trigger"?

**Prompt questions:**

What actions should the eTAPER tool trigger?

Who are the key people involved?

How might the eTAPER tool **facilitate communication** between patients and healthcare providers?

Discuss what the interaction should look like

What mode of review (virtual or face-to-face) is preferred?

**Establishing workflow and usage procedures**

(Discuss the workflow and usage procedures of the eTAPER tool.)

What should the workflow procedure for using the eTAPER tool look like?

**Prompt questions:**

When should the eTAPER tool be utilised in a patient's journey?

Should the tool adopt a reactive or proactive approach?

How frequently should indicators be run?

How often should the dashboard be checked?

Who should be responsible for using the tool at each stage?

What challenges exist in integrating the tool into existing workflows?

How can we support patients with special needs

**Supporting healthcare professionals to use the tool**

(Identify support mechanisms needed by healthcare professionals to use the eTAPER tool effectively.)

What support do healthcare professionals need to use the eTAPER tool effectively?

**Prompt questions:**

What training or education is necessary for healthcare professionals?

How can peer-to-peer support assist professionals?

What guidance materials would be helpful?

Are there any additional features/functions needed?

What concerns or barriers do professionals (and patients) foresee, and how can they be addressed?

What **challenges** do you foresee in implementing the eTAPER tool, and how can they be addressed?

**Envisioning Future Enhancements for the eTAPER Tool**

(Gather insights and suggestions for future enhancements of the eTAPER tool.)

What additional features should be included in future versions of the eTAPER tool?

**Prompt questions:**

What additional resources or materials should be integrated?

How can the user interface and experience be optimised?

Are there features from other tools that could enhance the eTAPER tool?

What technological features would enhance the tool's usability?

Are there **any additional features essential** for the eTAPER tool's effectiveness?

What features are essential for promoting patient engagement and adherence?

How can educational resources be integrated into the eTAPER tool?

**Closing**

Thank you everyone, I hope you have had some insightful discussions.

After today's experience-based co-design session concludes, I would like to provide you with a clear outline of the next steps to ensure that the insights gathered are effectively utilised in the development of the eTAPER tool.

We will review and analyse the notes, recordings, and key insights gathered from today's focus group discussions and identify common themes, recurring challenges, and solutions proposed by both patients and healthcare professionals.

Based on the insights obtained, we will begin revising prototype features for the eTAPER tool that address the identified needs and challenges. We will ensure that the prototype features are aligned with patient-centred principles and aim to optimise acute pain management and opioid use after surgery.

At the next meeting, we will share the revised prototype features from today's session with you for feedback and validation. We will further aim to incorporate any additional insights or suggestions provided by the group and discuss issues around the implementation of these tools in the future.

Thank you to each of you for your active participation and contributions today. Your input will be essential as we analyse the responses and draw conclusions.

Online supplement 4. Focus group schedule for the co-design workshop (2)

**Developing An Intervention to Optimise Acute Pain and Opioid Use After Surgery**

**Joint Patient and Healthcare Professional Focus Group Schedule – Session 4**

**Opening**

(Introduction, briefing the purposes of the focus group, set up the ground rules)

Good morning, everyone. Welcome to our fourth experience-based co-design session. I am delighted we will work together again today jointly as a team of patients and healthcare professionals to develop the eTAPER tool's features further.

***(Summary of the previous meeting)***

Before we start today's discussion, I would like to summarise critical themes emerging from last week's discussion. First, let's recap the prototype eTAPER tool 1.0 (slide 1).

Regarding the four main discussion points we discussed, the saturated themes around the current features of the draft prototype of eTaper included …. (slides 2-5). These results give us a good insight into how the eTAPER could work. I hope they captured the critical points that you raised last time.

***(Aim of this meeting)***

Today, we would like to discuss the eTAPER tool's draft prototype and how it can be implemented in clinical practice, considering the four main aspects (slide 6).

Following our discussion today, we will refine the draft prototype further and be ready to share the final output with you at the celebration/dissemination event.

***(Schedule and housekeeping)***

Like last week, to facilitate effective conversations, we will divide into two breakout rooms, each led by two facilitators.

Facilitators will help with the focus group discussions, take notes and manage the time.

If you need a break during the discussion, please inform facilitators using the chat box.

The facilitator will remain impartial throughout the event.

***(Confidentiality and anonymity of recording)***

Throughout our discussion, we will be recording audio.

Your honesty is paramount, and there are no right or wrong answers. Please be assured all conversations will be treated with utmost confidentiality and anonymity. Do not disclose identifiable information outside this focus group.

***(Ground rules)***

Before we begin, let's agree on some ground rules (same as last week):

We kindly ask that we take turns speaking to ensure a clear audio recording and allow others to express their opinions even if you disagree with them.

If you want to share your views, please use the '**raise your hand**' function and respect the discussion's confidentiality.

Please also express your views verbally to enable audio recording.

Thank you for being a part of this vital conversation. Your insights will make a real difference.

***(Split into breakout rooms)***

Now, we will split into two breakout rooms for the main discussion for **60 minutes**, and then we will get back together in the main room at **11:30** to wrap up our discussions today. See you back at **11:30**.

**Discussion**

Hello, and welcome to the discussion of this breakout room. My name is [XXXX], and I will facilitate the discussions. Together with me is [XXX] to help with this session. We have [XXXXXX] (name the participants) in this group.

**Ice breaking / Introduction**

I think it will be useful if we get to know each other in this group. Some of you have met. Could we go around the room introducing ourselves? For patients (name, type of surgery, current pain medication). For healthcare professionals (name and roles).

**About the usability**

How do we ensure and enhance the usability of the tool?

**Prompt questions:**

Do you have any other views about the eTaper's trigger actions (slide 3)?

Regarding the reactive action that we discussed at the last session, providing patients with an information leaflet when an alert is sent:

Do you have any suggestions on what information should be included in the leaflet?

How about the BPS leaflets? [Managing pain after your surgery](https://www.britishpainsociety.org/static/uploads/resources/files/pain_management_after_surgery_English.pdf); [understanding & managing pain after surgery](https://www.britishpainsociety.org/static/uploads/resources/files/Taster_for_web_Aug_2017.pdf)

How do we cater to non-English speaking patients?

How do we ensure the tapering plan is communicated to all those involved in patient care (physio, community pharmacists) to achieve the 'gold standard framework' we discussed last week (slide 4)?

What should the referral pathways for patients with special needs look like?

How can we develop clear referral pathways for patients with special needs?

**About usefulness**

How can we ensure the eTAPER tool is helpful in its users?

**Prompt questions:**

Is there any education or training needed for the users of the tool (patients and healthcare professionals)? If so, how do we deliver this? (slide 5)

Is there any training needed for healthcare professionals on tapering doses?

Is there a place for peer-to-peer review in 'complex' cases? E.g., MDT discussion within the GP practice to ensure a holistic review. Can this be incorporated into the tool?

Are there any additional functions/features needed to enhance the effectiveness of the tool further?

How do we ensure the sustainability of the tool and measure success on an ongoing basis?

**About the awareness**

How to raise awareness of the tool amongst patients/healthcare professionals?

**Prompt questions:**

What strategies can be employed to launch/communicate the tool with healthcare professionals and patients?

What strategies can be used to promote the tool to both groups

**About the accessibility**

How can the tool be made accessible to patients and healthcare professionals?

**Prompt questions:**

How can the tool be ensured to be easy to use and seamless to integrate into clinical practice? Should an alert also be sent to the community pharmacist?

How can we ensure the tool is accessible to patients with cultural and language barriers/healthcare professionals with busy schedules. Who should conduct the review? (*do we need to think of a risk assessment and prioritisation tool and review of patients based on their acuity levels?)*

How do we involve carers/family members?

**Closing**

Thank you, everyone, I hope you have had some insightful discussions.

In the previous session, we had the opportunity to discuss what we would like future iterations of eTAPER to look like (our 'wish list'). I want to share with you some of these discussions:

Better prepare patients in terms of realistic pain expectations (As Antony mentioned, nobody tells you about the pain and how to prepare beforehand)

Use patient waiting lists as an opportunity to prep patients on pain management and set realistic goals and expectations

As mentioned in the focus group last week, is there an opportunity for pre-surgical management?

Involving a psychologist as part of the referral options

Integrated care model with consultants being involved in the conversation, technology in place in hospitals with outpatients / pre-op clinics undertaking video consultations

This is an opportunity for pharmacists to visit patients at home to see how they are coping, provide tapering advice and report back to GPs. Explore services already commissioned to identify opportunities.

Thank you all for your commitment and energy to this project.

After the conclusion of today's experience-based co-design session, I would like to provide you with a clear outline of the following steps to ensure that the insights gathered are effectively utilised in the further refinement of the draft prototype.

Like last time, we will review and analyse the notes, recordings, and critical insights from today's focus group discussions. We will also identify common themes, recurring challenges, and solutions proposed by both patients and healthcare professionals.

At the final dissemination meeting, we will share the final version of eTAPER 1.0 with you from today's session and a plan for implementation for feedback. The original date was set as 17th May, but we must re-arrange this. I will send a doodle poll to show your availability for the dissemination event. We plan this event as an in-person meeting at the Health Innovation Manchester City labs venue for those who can attend and as an online option for those unable to meet in person. All travel and accommodation expenses will be covered,

A big thank you to each of you for your active participation and contributions today. Your input will be essential as we analyse the responses and draw conclusions.

Online supplement 5. Theoretical Domains Framework domain labels and

definitions

| **Domain** | **Definition** |
| --- | --- |
| 1. Knowledge | An awareness of the existence of something. |
| 2. Skills | An ability or proficiency acquired through practice. |
| 3. Social/professional role and identity | A coherent set of behaviours and displayed personal qualities of an individual in a social or work setting. |
| 4. Beliefs about capabilities | Acceptance of the truth, reality, or validity about an ability, talent, or facility that a person can put to constructive use. |
| 6.beliefs about consequences | Acceptance of the truth, reality, or validity about outcomes of a behaviour in a given situation. |
| 7. Reinforcement | Increasing the probability of a response by arranging a dependent relationship, or contingency, between the response and a given stimulus. |
| 8. Intentions | A conscious decision to perform a behaviour or a resolve to act in a certain way. |
| 9. Goals | Mental representations of outcomes or end states that an individual wants to achieve. |
| 10. Memory, attention and decision processes | The ability to retain information, focus selectively on aspects of the environment and choose between two or more alternatives. |
| 11. Environmental context and resources | Any circumstance of a person's situation or environment that discourages or encourages the development of skills and abilities, independence, social competence, and adaptive behaviour. |
| 12. Social influences | Those interpersonal processes that can cause individuals to change their thoughts, feelings, or behaviours. |
| 14. Behavioural regulation | Anything aimed at managing or changing objectively observed or measured actions. |

(Note) The definition is based on Cane et al. 2012.
