# Supplementary material for: Collaborative development of a digital intervention to support opioid tapering after surgery in primary care: an experience-based co-design study with patients and clinicians
Source: BMJ Open. 2026 Jun 30;16(6):e110623. doi: 10.1136/bmjopen-2025-110623 (PMC13331231; doi:10.1136/bmjopen-2025-110623)
Supplement: Supplementary file 2 [file bmjopen-2025-110623.reviewer_comments.pdf]

PEER REVIEW HISTORY

BMJ Open publishes all reviews undertaken for accepted manuscripts. Reviewers are asked to complete a checklist review form and are provided with free text boxes to elaborate on their assessment. These free text comments are reproduced below.

ARTICLE DETAILS

Title (Provisional)

Collaborative development of a digital intervention to support opioid tapering after surgery in primary care: an experience-based co-design study with patients and clinicians

Authors

Bansal, Neetu; Armitage, Chris; Hawkes, Rhiannon Eleri; Ashcroft, Darren; Chen, Li-chia

---

VERSION 1 - REVIEW

**Reviewer**	**1**
**Name**	**Edwards, Karlyn**
**Affiliation**	**University of Pittsburgh, Medicine**
**Date**	**24-Oct-2025**
**COI**	**None**

Introduction:

- The authors do a nice job of describing why an opioid taper tool is needed. However, given the significant reductions in opioid prescribing in the last several years, I was left wondering 1) how many patients are prescribed opioids following surgery, and 2) how many of those patients require management of opioid prescriptions by a PCP? I think this is needed to characterize the need and potential use of this tool.

- Define the acronym PINCER.

- use of the eTAPER tool seems to hinge on the use of the SMASH dashboard. Could the authors describe more about if and how this dashboard is already used in clinical practice?

Methods:

- There are many acronyms, could the authors spell out a few of them to increase readability?

- it would be helpful to briefly summarize findings from the prior paper describing the EBCD process. Did the prior study and the current one use the same patient participants?

Results:

-The authors state trust emerged as a central theme, however only a small paragraph with no quotations touched on this. Could the authors provide more information and quotes in this area? Given tightening regulations and the potential clinical and legal implications of overprescribing or misusing opioids, this seems like a very important area to address for a digital tool that monitors opioid use.

Discussion:

- A broader discussion of other digital tools to assist with opioid tapering is needed to help the reader understand how this tool is both similar and different from other tools that have been created and tested. It would also be helpful to know how the data collected through the co-design may contribute to novel features of the eTAPER tool that may not be reflected in other evidenced-based digital tools already available.

- Additionally, I think a discussion of how patient involvement in the design process may impact patient trust in their use and participation in this program is needed, in addition to how authors plan to address concerns related to patient trust in future iterations of the tool.

**Reviewer**	**2**
**Name**	**Grossman, Ellie**
**Affiliation**	**Cambridge Health Alliance**
**Date**	**02-Dec-2025**
**COI**	**None**

Review of ‘Collaborative development of a digital intervention to support opioid tapering after surgery in primary care: an experience-based co-design study with patients and clinicians’

Summary thought:

The authors present qualitative data that was part of a project to build a digital tool to help manage opioids and opioid tapering after surgery. In the Introduction, the authors state that their aim in this paper is to describe the findings from a set of patient and provider focus groups that elicited barriers and facilitators related to opioid tapering and discussed effective behavior change techniques. The ideas presented in this qualitative data may be informative for others interested in similar interventions, but the authors spend so much verbiage on other aspects of their project that the key findings related to their main purpose get a little buried. This manuscript would benefit from editing to remove some of this extraneous background information and enhance description of their key findings (perhaps with more direct quotations or other qualitative findings).

Major Essential Revisions:

• The authors state at the end of their Introduction that the focus of this manuscript is on the 2 focus groups that were part of the co-design process – and this matches to their description of the 'setting' and 'participants' in the abstract. However, the 'objectives' and 'design' sections of the abstract speak to a broader purpose – describing the entirety of their eTAPER tool design. The authors should be more careful in their choice for focus of this manuscript (i.e. entirety of eTAPER design process, or just the focus groups?), and then be consistent with this choice throughout the paper.

• As noted above, this paper's main weakness is its inconsistent focus. This is especially apparent in the Introduction, which is lengthy and describes much background related to opioid tapering and digital tools like the SMASH. If the focus of this manuscript is on the qualitative findings from focus groups with patients and health-care providers on how to optimize postoperative opioid management and brainstorm about a digital tool, then much of the Introduction can probably be omitted – which will better prepare the reader for the meat of their findings. The Discussion has a similarly meandering and unfocused structure and could use similar editing/refocus.

• I worry about potential loss of anonymity for participants, especially the health-care professionals, since there is so much detail provided about professional role and other characteristics in the related Table – and then individual quotations are labeled with participant IDs. I suspect the information in the 2 tables describing patient and provider participants could be summarized in text, and thereby omit the level of detail in these 2 tables.

• It is somewhat difficult to follow the key qualitative findings because they are buried in long texty paragraphs. I suspect the results might be more easily digested if more of them were presented in bullet points and/or tables/figures.

• The authors should more clearly explain why they chose these specific health-care professionals for the focus groups. Most of the participants are pharmacists and/or administrators, while in most settings one might imagine that postoperative prescribing would be led by surgeons or primary-care providers – who seem under-represented (or not represented at all, for surgeons) in the focus groups.

Minor Essential Revisions:

• In every instance where the entirety of the eTAPER design process is described (e.g. online supplement 1, Figure 2), the authors should note which segment of the design process is reflected in this paper.

• The use of a 'coding tree' is fairly standard in qualitative studies. The authors should explain why they did not use one in their project (online supplement 2).

• Similarly, it is unclear why they say they omitted mention of some characteristics of the main researcher (online supplement 2) – but they do seem to include this information in the paper (page 11, 'researcher positioning').

• I am not clear on the purpose of Figure 1 or how it is linked to the text which refers to it (bottom of page 7).

• The authors mention use of 'breakout rooms' in the focus groups – which makes it sound like there were multiple smaller-group discussions going on within each session. The authors should clarify how these 'breakout rooms' were formed and managed, and what their purpose was. If the purpose was just to split the N of 20 people into smaller groups of 10 that happened to run simultaneously, then the authors should probably consider each session as 2 focus groups per topic – for a total of 4 focus groups described in this study.

• The authors should explain more about why they chose the Theoretical Domains Framework (TDF) to categorize their qualitative findings.

• The authors have 4 tables which are all labeled as 'table 1.' They should be renumbered.

• It is unclear why patients are labeled as 'British English' for ethnicity, while healthcare professionals are labeled as 'white British.' If these are the same, they should be labeled the same – and if they are different, then the labels should be defined somewhere.

Reviewer **3**
Name **Liauw, Winston**
Affiliation **University of New South Wales Saint George and Sutherland Clinical School**
Date **20-Jan-2026**
COI **None**

---

this is an important area of investigation

clearly the major limitation is the overall small sample size in the context of qualitative work. further validation is required

---

VERSION 1 - AUTHOR RESPONSE

Response to Reviewer 1 comments

Comment:
The authors do a nice job of describing why an opioid taper tool is needed. However, given the significant reductions in opioid prescribing in the last several years, I was left wondering 1) how many patients are prescribed opioids following surgery, and 2) how many of those patients require management of opioid prescriptions by a PCP? I think this is needed to characterize the need and potential use of this tool.
Response:

Thank you for this helpful comment. We agree that quantifying the extent of post-operative opioid prescribing and subsequent primary care management is important to contextualise the need for the intervention. We have now revised the Introduction to include additional evidence on the prevalence of opioid prescribing following surgery and the extent to which ongoing management occurs in primary care

Lines [92-102]

Although opioid analgesics are generally indicated for only a short duration post-surgery, many patients continue to use them for extended periods (1, 6-8). Several studies have highlighted concerns regarding excessive opioid prescribing at hospital discharge following surgery. A large multicentre prospective cohort study reported that approximately 30% of patients were prescribed opioids at discharge, and that patients were prescribed more than twice the quantity of opioids they actually consumed within the first seven days post-discharge(2). Similarly, Lobo et al., in a retrospective study across fourteen NHS Trusts, found that 21% of patients were prescribed opioids following major surgery (9). These findings demonstrate that a substantial proportion of patients continue to receive opioids after discharge, often in quantities exceeding clinical need, highlighting an important opportunity for intervention.

Comment:

Define the acronym PINCER

Response:

We have now defined the acronym PINCER at first use in the manuscript.

Lines [113-114]

SMASH has been instrumental in identifying patients at risk of hazardous prescribing based on the nationally recognised PINCER (pharmacist led information technology intervention for reducing clinically important errors) indicators (18, 19).

Comment:

Use of the eTAPER tool seems to hinge on the use of the SMASH dashboard. Could the authors describe more about if and how this dashboard is already used in clinical practice?

Response:

We agree that providing additional context on the use of the SMASH dashboard in clinical practice is important. We have now expanded the manuscript to describe how SMASH is currently implemented in primary care.

Lines [119-129]

The Safety Medication Dashboard (SMASH) is a pharmacist-led, web-based audit and feedback tool that is currently implemented within primary care settings(18). It uses routinely collected electronic health record data to identify patients at risk of hazardous prescribing, based on validated safety indicators such as those defined by the PINCER indicators(18). The dashboard supports general practice teams, particularly clinical pharmacists, in prioritising medication reviews and optimising prescribing. The eTAPER intervention has been developed to integrate within this existing infrastructure, extending its functionality to identify patients prescribed opioids following surgery and to support timely review and tapering in primary care.

Comment:

There are many acronyms, could the authors spell out a few of them to increase readability?

Response:

We agree that the use of multiple acronyms may affect readability. We have now revised the manuscript to ensure that all acronyms are defined in full at first use and have reduced the use of less essential abbreviations where possible to improve clarity for the reader.

Comment:

It would be helpful to briefly summarize findings from the prior paper describing the EBCD process. Did the prior study and the current one use the same patient participants?

Response:

We agree that providing additional context on the earlier phases of the EBCD process would improve clarity. We have now included a summary of the key findings from the earlier study, which identified important barriers to opioid tapering following surgery, including challenges in communication across care settings, lack of clear tapering guidance, and limited patient support after discharge. These findings informed the initial development of the eTAPER intervention. We have also clarified that the

same patient participants were involved across both phases of the EBCD process, ensuring continuity and enabling iterative refinement of the intervention based on their lived experiences.
Lines [135-139]
This study builds on earlier phases of the Experience-Based Co-Design (EBCD) process, which identified key barriers to opioid tapering after surgery, including fragmented communication between care settings, lack of clear tapering guidance, and limited support for patients following discharge(24). These findings informed the initial development of the eTAPER intervention. In the present study, we undertook the final co-design phase to refine the intervention. The same patient participants were involved across both phases, allowing continuity and iterative refinement based on their experiences.

Comment:
The authors state trust emerged as a central theme, however only a small paragraph with no quotations touched on this. Could the authors provide more information and quotes in this area? Given tightening regulations and the potential clinical and legal implications of overprescribing or misusing opioids, this seems like a very important area to address for a digital tool that monitors opioid use.
Response:
We agree that trust is a critical theme, particularly in the context of increasing regulatory scrutiny and the clinical and legal responsibilities associated with opioid prescribing. We have now expanded the results section to provide a more in-depth exploration of the theme of trust, supported by additional participant quotations. This expanded analysis highlights how trust operates at multiple levels, including the patient–clinician relationship, trust in digital tools, and clinicians' concerns regarding professional autonomy and accountability.
Lines [409-439]

Comment:
A broader discussion of other digital tools to assist with opioid tapering is needed to help the reader understand how this tool is both similar and different from other tools that have been created and tested. It would also be helpful to know how the data collected through the co-design may contribute to novel features of the eTAPER tool that may not be reflected in other evidenced-based digital tools already available.
Response:
We agree that situating the eTAPER intervention within the context of existing digital tools is important to highlight its contribution. We have now expanded the Discussion to include a broader overview of existing digital and technology-enabled interventions designed to support opioid tapering and prescribing optimisation. We have also clarified how eTAPER both aligns with and differs from these approaches.
Lines [489-499]
Digital interventions to support opioid optimisation and tapering are increasingly being developed, including patient-facing applications, electronic prescribing alerts, and clinical decision support tools (35, 36). Many of these interventions focus on discrete components of care, such as patient education, pain self-management, or prescribing alerts, and are often implemented either at the point of prescribing or as standalone tools. In contrast, the eTAPER intervention has been designed as a clinician-facing, primary care–integrated tool embedded within an existing prescribing safety platform (SMASH). This enables identification of patients prescribed opioids following surgery and supports timely review and tapering within routine clinical workflows. Notably, eTAPER specifically targets the transition between secondary and primary care, a recognised high-risk period for ongoing opioid use that is often not addressed by existing digital tools.

Comment:
Additionally, I think a discussion of how patient involvement in the design process may impact patient trust in their use and participation in this program is needed, in addition to how authors plan to address concerns related to patient trust in future iterations of the tool.

Response:
We have now expanded the discussion section to highlight how the use of an Experience-Based Co-Design (EBCD) approach may enhance patient trust by ensuring that the intervention reflects patient priorities, concerns, and lived experiences. We have added a plan to address concerns related to patient trust in future iterations of the tool .

Lines [500-514]

Patient involvement in the co-design process also has important implications for trust, which is a critical determinant of engagement with digital health interventions. By incorporating patient perspectives throughout development, eTAPER was shaped to reflect patient priorities, concerns and lived experiences, which may enhance transparency, perceived relevance and trust in the tool. This is particularly important in the context of opioid tapering, where patients may be sensitive to perceived reductions in medication or concerns about being monitored or restricted.

However, trust may be undermined if digital tools are perceived as replacing clinical judgement or acting as surveillance mechanisms. Future iterations of eTAPER should therefore prioritise clear communication about the purpose of the tool, emphasising its role in supporting, rather than directing, clinical decision-making. Enhancing continuity of care, enabling personalised tapering plans, and ensuring that clinicians retain flexibility to adapt recommendations will be essential. Ongoing co-refinement with patients and healthcare professionals, alongside transparent messaging and training, will be key to strengthening trust and supporting sustained engagement with the intervention.

Response to Reviewer 2 comments

Comment:

The authors present qualitative data that was part of a project to build a digital tool to help manage opioids and opioid tapering after surgery. In the Introduction, the authors state that their aim in this paper is to describe the findings from a set of patient and provider focus groups that elicited barriers and facilitators related to opioid tapering and discussed effective behavior change techniques. The ideas presented in this qualitative data may be informative for others interested in similar interventions, but the authors spend so much verbiage on other aspects of their project that the key findings related to their main purpose get a little buried. This manuscript would benefit from editing to remove some of this extraneous background information and enhance description of their key findings (perhaps with more direct quotations or other qualitative findings).

Response:

Thank you for this helpful feedback. We have revised the manuscript to improve focus and clarity by streamlining background content and strengthening the presentation of the qualitative findings. We have also included additional participant quotations and improved the structure of the Results section to better highlight key barriers, facilitators, and behaviour change techniques.

Comment:

The authors state at the end of their Introduction that the focus of this manuscript is on the 2 focus groups that were part of the co-design process – and this matches to their description of the 'setting' and 'participants' in the abstract. However, the 'objectives' and 'design' sections of the abstract speak to a broader purpose – describing the entirety of their eTAPER tool design. The authors should be more careful in their choice for focus of this manuscript (i.e. entirety of eTAPER design process, or just the focus groups?), and then be consistent with this choice throughout the paper.

Response

We agree that the focus of the manuscript was not sufficiently consistent across sections. In response, we have clarified abstract to focus specifically on the focus groups conducted as part of the co-design process.

Comment:

As noted above, this paper's main weakness is its inconsistent focus. This is especially apparent in the Introduction, which is lengthy and describes much background related to opioid tapering and digital tools like the SMASH. If the focus of this manuscript is on the qualitative findings from focus groups with patients and health-care providers on how to optimize postoperative opioid management and brainstorm about a digital tool, then much of the Introduction can probably be omitted – which will better prepare the reader for the meat of their findings. The Discussion has a similarly meandering and unfocused structure and could use similar editing/refocus.

Response

We agree that the manuscript would benefit from a clearer and more consistent focus. In response, we have substantially streamlined the Introduction to remove extraneous background content and

better align it with the qualitative focus of this paper. We have also revised the Discussion to improve structure and coherence, ensuring it remains closely aligned with the study findings and key themes.

Comment:
I worry about potential loss of anonymity for participants, especially the health-care professionals, since there is so much detail provided about professional role and other characteristics in the related Table – and then individual quotations are labeled with participant IDs. I suspect the information in the 2 tables describing patient and provider participants could be summarized in text, and thereby omit the level of detail in these 2 tables.
Response:
Thank you for raising this important point. We take participant confidentiality seriously. In response, we have revised the presentation of participant characteristics in the tables by removing columns such as type of surgery for patients and clinical role for healthcare participants to protect participant confidentiality.

Comment:
It is somewhat difficult to follow the key qualitative findings because they are buried in long texty paragraphs. I suspect the results might be more easily digested if more of them were presented in bullet points and/or tables/figures.
Response:
Thank you for this helpful suggestion. In response, we have revised the Results section to improve clarity and readability by reducing dense text and presenting key findings more concisely. Where appropriate, we have incorporated structured formatting to better highlight themes and make the findings easier to follow.
We believe these changes improve the accessibility and overall presentation of the qualitative results.

Comment:
The authors should more clearly explain why they chose these specific health-care professionals for the focus groups. Most of the participants are pharmacists and/or administrators, while in most settings one might imagine that postoperative prescribing would be led by surgeons or primary-care providers – who seem under-represented (or not represented at all, for surgeons) in the focus groups.
Response:
Thank you for this important observation. We have revised the manuscript to more clearly justify the selection of participants for the focus groups. Specifically, we explain the rationale for including pharmacists and administrators, given their key roles in medicines optimisation, implementation, and post-discharge care within the study context. We also acknowledge the under-representation of surgeons and highlight this as a limitation, noting how future work will seek to incorporate a broader range of stakeholders, including surgical and primary care perspectives.
Lines [193-202]
Healthcare professionals were selected to reflect those most involved in ongoing opioid prescribing and tapering following hospital discharge, particularly within primary care settings where responsibility for pain management is often transferred. Pharmacists, including those working in general practice, were purposively included due to their central role in medicines optimisation and their interaction with prescribing safety platforms such as SMASH, within which the eTAPER intervention is embedded. While surgeons play an important role in initiating post-operative opioid prescribing, their involvement in longer-term opioid management is typically limited; therefore, the study prioritised professionals involved in continuity of care after discharge. Nevertheless, anaesthetists were included to provide perspectives from secondary care pain management.

Comment:
In every instance where the entirety of the eTAPER design process is described (e.g. online supplement 1, Figure 2), the authors should note which segment of the design process is reflected in this paper.
Response:
We have revised Figure 2 to include a note that this paper focuses on the results from sessions 3-5

Comment:
The use of a ‘coding tree’ is fairly standard in qualitative studies. The authors should explain why they did not use one in their project (online supplement 2).
Response

While coding trees are commonly used in qualitative research to illustrate hierarchical theme development, a traditional coding tree was not presented in this study. This is because the analysis was guided by the Theoretical Domains Framework (TDF), which provided a pre-defined structure for organising codes into theoretically informed domains. Rather than developing a purely inductive hierarchical coding framework, codes were iteratively mapped to relevant TDF domains and linked to behaviour change techniques (BCTs). As such, findings are presented in relation to these domains which was considered a more appropriate and meaningful representation of the analysis in the context of theory-informed intervention development.
We have added a note to Online supplement explaining why the coding tree was not used

Comment:
Similarly, it is unclear why they say they omitted mention of some characteristics of the main researcher (online supplement 2) – but they do seem to include this information in the paper (page 11, 'researcher positioning').
Response:
We have now corrected this in Online supplement 2 to reflect sections in the manuscript where the main researcher's information is noted

Comment:
I am not clear on the purpose of Figure 1 or how it is linked to the text which refers to it (bottom of page 7).
Response:
We have deleted Figure 1 as on reflection it does not add value to the paper. Figure 2 has been renamed to Figure 1 and ensures it links appropriately to the text

Comment:
The authors mention use of 'breakout rooms' in the focus groups – which makes it sound like there were multiple smaller-group discussions going on within each session. The authors should clarify how these 'breakout rooms' were formed and managed, and what their purpose was. If the purpose was just to split the N of 20 people into smaller groups of 10 that happened to run simultaneously, then the authors should probably consider each session as 2 focus groups per topic – for a total of 4 focus groups described in this study.
Response:
Thank you for this helpful observation. We have revised the manuscript to clarify how the breakout rooms were organised and their purpose within the focus groups.
Lines [216-226]
Given the relatively large number of participants per session, participants were divided into smaller breakout rooms (typically 4–6 participants per group) to facilitate more in-depth discussion and ensure all participants had the opportunity to contribute. Breakout rooms were purposively composed to include a mix of patients and healthcare professionals, and each room was supported by a facilitator to guide discussion and ensure consistency across groups.
The purpose of the breakout rooms was to enhance interaction and depth of discussion rather than to conduct separate focus groups. Following the breakout discussions, participants reconvened in a plenary session where key points from each group were shared and discussed collectively, allowing for comparison and refinement of ideas across groups. Therefore, each workshop was treated analytically as a single co-design session rather than multiple independent focus groups.

Comment:
The authors should explain more about why they chose the Theoretical Domains Framework (TDF) to categorize their qualitative findings.
Response:
We have expanded the analysis section to include justification for using the theoretical domains framework to categorise our qualitative findings.
Lines [246-252]
The TDF was selected as it provides a comprehensive, theory-informed framework that synthesises constructs from multiple behaviour change theories into a set of domains relevant to understanding healthcare behaviours (26). Its use is well established in implementation research and is particularly suited to identifying determinants of complex behaviours, such as opioid prescribing and tapering across care transitions. Applying the TDF enabled the research team to move beyond descriptive

themes and systematically interpret the underlying behavioural mechanisms influencing both patient and clinician actions.

Comment:
The authors have 4 tables which are all labelled as ‘table 1.’ They should be renumbered.
Response:
Thank you for highlighting this error. We have corrected the table numbering throughout the manuscript to ensure each table is accurately labelled.

Comment:
It is unclear why patients are labelled as ‘British English’ for ethnicity, while healthcare professionals are labelled as ‘white British.’ If these are the same, they should be labelled the same – and if they are different, then the labels should be defined somewhere.
Response:
Thank you for highlighting this inconsistency. We have revised Tables 2 and 3 to ensure consistent terminology is used when reporting ethnicity across participant groups.

Response to Reviewer 3 comments

Comment:
This is an important area of investigation, clearly the major limitation is the overall small sample size in the context of qualitative work. further validation is required.
Response:
Thank you for this important comment. We acknowledge the relatively small sample size in this study and have noted this as a limitation in the discussion section. We also emphasise that, as a qualitative study within a co-design process, the aim was to generate in-depth insights rather than achieve generalisability.
Lines [548-557]
However, several limitations should be considered. The relatively small and heterogeneous sample, comprising eight post-surgical patients and twelve clinicians, may limit the representativeness of the findings, although the insights generated are likely to be transferable to

VERSION 2 - REVIEW

**Reviewer**	**1**
**Name**	**Edwards, Karlyn**
**Affiliation**	**University of Pittsburgh, Medicine**
**Date**	**24-Apr-2026**
**COI**	

The authors did an exceptional job addressing reviewers comments. I have no further suggestions or revisions.

**Reviewer**	**2**
**Name**	**Grossman, Ellie**
**Affiliation**	**Cambridge Health Alliance**
**Date**	**10-May-2026**

COI

Review of 'Collaborative development of a digital intervention to support opioid tapering after surgery in primary care: an experience-based co-design study with patients and clinicians – Revision 1'

Summary thought:
The authors have done an acceptable job of responding to previous comments. The focus of this manuscript is more clear, as is its connection to a larger project and role within the health-care system. However, given its relatively narrow generalizability (i.e. most patients do not get long-term opioids after surgery), I suspect it could be condensed to a shorter 'research report' (with graphical attention drawn to participant quotations and themes raised) rather than a full-length manuscript. If this manuscript does get cut down for brevity, I suspect that much of the background information about the eTAPER tool and SMASH tools could get distilled into 2 sentences along the lines of 'We were building an electronic tool to sit alongside the electronic medical record and provide assistance to PCPs in managing postoperative opioid prescriptions. Toward that end, we held 2 focus groups including patients and health-care professionals to evaluate draft versions of such a tool. In this manuscript, we share themes that emerged from these discussions.'

Essential Revisions:

- The authors spend a lot of time/text situating their work inside a larger co-design project. Although they have made their narrow focus clearer in this revision, the scope for this manuscript remains somewhat unclear. For example, in the Abstract the authors state that the setting is 2 focus groups and 1 inperson stakeholder event. However, all of the data they present seems to come from the patients or health-care providers who attended the 2 focus groups. It is unclear what role the inperson stakeholder event had; if it did not provide data that was analyzed and included in this manuscript, likely mention of it can be omitted. (And if there was data from this event that was included in the manuscript, then the authors need to provide more information about the size of this event, who attended, and the manner in which qualitative data was collected at a large celebration.)
- The authors need to add a bit more detail to description of the eTAPER tool and SMASH dashboard, which seem to be linked to the electronic medical record. Are either of these tools patient-facing, and if so, in what way? (And if they are not patient-facing, why is it important to have patients weigh in on their design?)
- The authors describe the eTAPER intervention as being embedded in the SMASH dashboard, and it sounds like these tools are used by pharmacists in primary care to review patient prescriptions and identify patients where intervention is needed. Do these pharmacists feed suggestions to the frontline PCPs? It would be interesting to understand a little more about how exactly these tools fit into routine care – for example, does this tool get used at the point of an opioid being prescribed in outpatient care? Or after an opioid is prescribed, but perhaps before a refill is given? Or something else?

VERSION 2 - AUTHOR RESPONSE

Response to Reviewer 1 comments

Reviewer: 1
Dr. Karlyn Edwards, University of Pittsburgh
Comments to the Author:
The authors did an exceptional job addressing reviewers comments. I have no further suggestions or revisions.
Response:
We thank the reviewer for their positive feedback and thoughtful review of our manuscript. We appreciate the acknowledgement of the revisions made in response to the reviewer comments

Response to Reviewer 2 comments
Reviewer: 2
Dr. Ellie Grossman, Cambridge Health Alliance
Comments to the Author:
see attached file

Comment:
The authors have done an acceptable job of responding to previous comments. The focus of this manuscript is more clear, as is its connection to a larger project and role within the health-care system. However, given its relatively narrow generalizability (i.e. most patients do not get long-term opioids after surgery), I suspect it could be condensed to a shorter 'research report' (with graphical attention drawn to participant quotations and themes raised) rather than a full-length manuscript.
Response:
We thank the reviewer for their thoughtful comments and for acknowledging the improvements made to clarify the focus of the manuscript. We agree that the study addresses a specific patient population; however, we believe the findings remain important given the significant health implications associated with long-term opioid use after surgery, despite affecting a smaller proportion of patients.

We have carefully considered the suggestion to condense the manuscript into a shorter research report. However, we feel that the current manuscript format allows sufficient detail to fully present the qualitative findings and stakeholder perspectives that informed the development of the intervention. We have reviewed the manuscript to improve conciseness where possible and have ensured that key participant quotations and themes are clearly highlighted throughout the results section.

Comment:
If this manuscript does get cut down for brevity, I suspect that much of the background information about the eTAPER tool and SMASH tools could get distilled into 2 sentences along the lines of 'We were building an electronic tool to sit alongside the electronic medical record and provide assistance to PCPs in managing postoperative opioid prescriptions. Toward that end, we held 2 focus groups including patients and health-care professionals to evaluate draft versions of such a tool. In this manuscript, we share themes that emerged from these discussions.'
Response:
We have substantially condensed the background description of the eTAPER and SMASH platforms to improve brevity and maintain focus on the qualitative findings presented in the manuscript. The revised section now provides a concise overview of the intervention and its purpose within primary care opioid management [Lines 124-128]:
"To address this challenge, the eTAPER intervention was developed for integration within the Safety Medication Dashboard (SMASH), a pharmacist-led electronic audit and feedback platform used in primary care to support medication safety reviews [18]. The intervention was designed to identify patients prescribed opioids following surgery and prompt timely review and opioid tapering in primary care, rather than at the point of initial prescribing"

Comment:
The authors spend a lot of time/text situating their work inside a larger co-design project. Although they have made their narrow focus clearer in this revision, the scope for this manuscript remains somewhat unclear. For example, in the Abstract the authors state that the setting is 2 focus groups and 1 inperson stakeholder event. However, all of the data they present seems to come from the patients or health-care providers who attended the 2

focus groups. It is unclear what role the inperson stakeholder event had; if it did not provide data that was analyzed and included in this manuscript, likely mention of it can be omitted. (And if there was data from this event that was included in the manuscript, then the authors need to provide more information about the size of this event, who attended, and the manner in which qualitative data was collected at a large celebration.)
Response:
Thank you for this important comment. We agree that the role of the in-person stakeholder event was not sufficiently clear in the original manuscript and may have led to confusion regarding the data sources underpinning this study.
To clarify, no primary qualitative data were collected or analysed during the in-person stakeholder engagement event. This event formed part of the broader Experience-Based Co-Design (EBCD) methodology and was conducted to support stakeholder engagement, present and validate emerging findings from the co-design workshops. It therefore served an important role in sense-checking but was not a data collection activity included in the formal analysis.
All qualitative data reported in this manuscript derive solely from the two online co-design workshops with patients and healthcare professionals. We have revised the manuscript accordingly to make this explicit in the Abstract and to ensure that only the data from the focus groups are described as contributing to the analysed findings. [Lines 43-48]:
Qualitative co-design study using two online co-design workshops and one in-person stakeholder engagement event guided by the Experience-Based Co-Design (EBCD) methodology and mapped to the Theoretical Domains Framework (TDF). Qualitative data were collected during the two online co-design workshops, while the in-person stakeholder engagement event was used to sense-check and validate the findings from the workshops. No new data were collected during the stakeholder engagement event.

Comment:
The authors need to add a bit more detail to description of the eTAPER tool and SMASH dashboard, which seem to be linked to the electronic medical record. Are either of these tools patient-facing, and if so, in what way? (And if they are not patient-facing, why is it important to have patients weigh in on their design?)
Response:
Thank you for this helpful comment, which allows us to clarify the nature and purpose of the eTAPER tool and SMASH dashboard and the rationale for patient involvement.
Neither the eTAPER tool nor the SMASH dashboard is directly patient-facing. Both are clinician-facing digital decision support tools designed to be embedded within or alongside the electronic medical record to support healthcare professionals in reviewing and managing post-operative opioid prescriptions in primary care.
Although these tools are not accessed directly by patients, we actively involved patients in their co-design because their lived experience of post-surgical opioid use is essential to ensuring that the tools reflect patient needs and expectations. In particular, patient input helped shape how tapering advice is framed, how safety-netting and reassurance are communicated, and how clinicians can better support shared decision-making and patient understanding during opioid reduction. We have now revised the manuscript to make this clearer by explicitly stating that the tools are clinician-facing and by strengthening the rationale for patient involvement within the co-design process. Added to Introduction [lines 128-133]:
“The eTAPER intervention is a clinician-facing digital decision support tool embedded within the SMASH platform and is not directly accessible to patients. Patients were involved in the co-design process to ensure the tool reflects lived experience of postoperative opioid use and to optimise how tapering advice is communicated in clinical consultations. Their input helped enhance acceptability, clarity and support for shared decision-making.”

Comment:
The authors describe the eTAPER intervention as being embedded in the SMASH dashboard, and it sounds like these tools are used by pharmacists in primary care to review patient prescriptions and identify patients where intervention is needed. Do these pharmacists feed suggestions to the frontline PCPs? It would be interesting to understand a little more about how exactly these tools fit into routine care – for example, does this

tool get used at the point of an opioid being prescribed in outpatient care? Or after an opioid is prescribed, but perhaps before a refill is given? Or something else

Response:

Thank you for this helpful comment. The SMASH dashboard with embedded eTAPER is used by primary care–based pharmacists as part of routine medicines optimisation and audit/feedback activity, rather than at the point of prescribing. Patients are identified after discharge when opioids initiated in secondary care are continued in primary care. Pharmacists then use eTAPER to support structured medication review and either initiate or more commonly communicate tapering recommendations to the responsible GP to support shared decision-making.

The tool is therefore positioned for post-prescribing review and tapering support during ongoing primary care management, not at the initial prescribing stage. We have clarified this workflow in the revised manuscript. Added to lines [126-128]:

“The intervention is designed to identify patients prescribed opioids following surgery and prompt timely review and opioid tapering in primary care, rather than at the point of initial prescribing.”

Comment **Note from Editor**

Reviewer 2 has provided some additional essential revisions, however, please note that we do not expect the authors to the rewrite the manuscript as a ‘research report’, but we have included this comment for transparency

Response:

We thank the Editor for this clarification. We confirm that we have retained the manuscript as a qualitative co-design intervention development study and have not revised it into a full research report. Revisions have focused on improving clarity based on the feedback.
